# Beyond Bacteria: A Study of the Enteric Microbial Consortium in Extremely Low Birth Weight Infants

**DOI:** 10.1371/journal.pone.0027858

**Published:** 2011-12-08

**Authors:** Mariam Susan LaTuga, Joseph Christopher Ellis, Charles Michael Cotton, Ronald N. Goldberg, James L. Wynn, Robert B. Jackson, Patrick C. Seed

**Affiliations:** 1 Department of Pediatrics, Albert Einstein College of Medicine, New York, New York, United States of America; 2 Department of Biology, Duke University, Durham, North Carolina, United States of America; 3 Department of Pediatrics, Duke University Medical Center, Durham, North Carolina, United States of America; 4 Nicholas School of the Environment and Center on Global Change, Duke University, Durham, North Carolina, United States of America; 5 Center for Microbial Pathogenesis, Duke University, Durham, North Carolina, United States of America; 6 Jean and George Brumley, Jr Neonatal-Perinatal Research Institute, Duke University, Durham, North Carolina, United States of America; Loyola University Medical Center, United States of America

## Abstract

Extremely low birth weight (ELBW) infants have high morbidity and mortality, frequently due to invasive infections from bacteria, fungi, and viruses. The microbial communities present in the gastrointestinal tracts of preterm infants may serve as a reservoir for invasive organisms and remain poorly characterized. We used deep pyrosequencing to examine the gut-associated microbiome of 11 ELBW infants in the first postnatal month, with a first time determination of the eukaryote microbiota such as fungi and nematodes, including bacteria and viruses that have not been previously described. Among the fungi observed, *Candida sp.* and *Clavispora sp*. dominated the sequences, but a range of environmental molds were also observed. Surprisingly, seventy-one percent of the infant fecal samples tested contained ribosomal sequences corresponding to the parasitic organism *Trichinella.* Ribosomal DNA sequences for the roundworm symbiont *Xenorhabdus* accompanied these sequences in the infant with the greatest proportion of *Trichinella* sequences. When examining ribosomal DNA sequences in aggregate, *Enterobacteriales, Pseudomonas, Staphylococcus,* and *Enterococcus* were the most abundant bacterial taxa in a low diversity bacterial community (mean Shannon-Weaver Index of 1.02±0.69), with relatively little change within individual infants through time. To supplement the ribosomal sequence data, shotgun sequencing was performed on DNA from multiple displacement amplification (MDA) of total fecal genomic DNA from two infants. In addition to the organisms mentioned previously, the metagenome also revealed sequences for gram positive and gram negative bacteriophages, as well as human adenovirus C. Together, these data reveal surprising eukaryotic and viral microbial diversity in ELBW enteric microbiota dominated bytypes of bacteria known to cause invasive disease in these infants.

## Introduction

Prematurity is the leading cause of neonatal deaths and long-term infant disability, with the rate of preterm births continuing to rise [1], [2]. Despite improved medical and respiratory management, the mortality rate for the most premature infants remains high [3], [4]. Extremely low birth weight infants (ELBW) are at increased risk for complications such as sepsis, meningitis, necrotizing enterocolitis (NEC) and poor growth- problems, all associated with high risk for neurodevelopmental impairment, and all of which may be impacted by the microbial communities in their gut [4], [5], [6], [7], [8], [9], [10]. Among premature infants, frequently used treatments, such as antibiotics and histamine-2 blockers are associated with an increased risk of necrotizing enterocolitis (NEC) and may exert their influence via alternations in gut microbiota [11], [12] .

In full term infants, using high-resolution molecular technologies, bacterial succession starts with a limited number of aerobic and anaerobic organisms and diversifies over the first postnatal year to assume a more adult makeup and distribution[13]. In comparison, in premature infants, the intestinal microbiome has been studied primarily using stool cultures and low resolution molecular techniques, for example DGGE, which may underestimate bacterial diversity [14], [15], [16], [17], [18], [19].

Three recent studies that applied high-throughput sequencing were primarily motivated by the hypothesis that deeper molecular analysis of the gut-associated microbiota of preterm infants would reveal previously unappreciated bacterial taxa or groups of taxa associated with NEC or systemic inflammatory response (SIRS). In the study by Mshvildadze, *et al.,* 10 predominant families of bacteria were identified among 12 infants with no specific association between the microbiota present and the development of NEC or SIRS [20]. The bacterial taxa were similar to full term infants at birth, with a dominance of *Enterobacteriaceae, Staphylococcaceae,* and *Lactobacilliales* (*Enterococcus* genus). In a study by Wang, *et al*, terminal restriction fragment length polymorphism analysis was employed to compare the bacterial constituents of the fecal microbiomes among infants with (N = 10) and without NEC (N = 10), with slightly lower diversity observed among infants with NEC[21]. Another recent study that examined one premature infant over the first three weeks of life found a predominance of limited bacterial genera and evidence of bacteriophage [22].

Previous studies have provided important insights into the bacterial constituents of the preterm gut microbiome but have not comprehensively evaluated for eukaryotic microbes and human viruses [16], [23], [24]. Invasive infections with *Candida* species cause significant morbidity and mortality among ELBW infants [25], [26]. Furthermore, ELBW infants consistently receive empirical anti-bacterial agents, which increase risk for invasive disease by *Candida sp* [25], [27], [28]. It is unclear how *Candida* species contribute to risk of invasive disease. They may invade primarily, infect once physiologic barriers have been breached or occupy the gut in the absence of healthy bacterial communities [29], [30], [31]. Although *Candida* species have been identified in the first two weeks of life in preterm infants using culture methods, a more complex analysis of primary succession of fungal, viral, and parasitic organisms in the ELBW infant gut-associated microbiome has not been performed.

Because of the critical role of development in the first month of life and the high risk of morbidity and mortality of ELBW infants, we applied current molecular methodologies to help resolve the microbial constituents of the gut-associated microbiome. Our study highlights unprecedented early fungal diversity, evidence of roundworms, human and bacterial viruses, and a bacterial community harboring many potential pathogens.

## Results

### Clinical characteristics

Of twenty-one infants eligible, eleven infants were enrolled in the study. The mean gestational age was 27 weeks, with an average birth weight of 765 gm (Table 1). All infants received antibacterials as well as oral nystatin as prophylaxis against *C.albicans* infection. Infants were fed maternal breast milk or donor breast milk with mean initiation and cumulative feeding days of 9 and 23, respectively (Table 2 and Information S2). Sample collection was convenience-based, with two samples collected from 8 infants and one collected from the remaining three.

**Table 1:** Demographic data for the clinical cohort.

Infant	GA (wk)	Gender	BW (gm)	Delivery mode	Bacteremia	Other	Death	Sample collection (day of life)
1	26	M	925	Vaginal	CONS		No	30
2	25	F	720	Caesarean section^1^	CONS	SVT	No	25
3	24	M	490	Caesarean section^2^	CONS	Hypospadias	No	21, 36
4	27	M	900	Vaginal		Hypertension	No	25, 25
5	25	M	980	Vaginal		PDA ligation	No	20, 34
6	27	F	882	Caesarean section^3^	CONS	Heart block- mat. Lupus	Yes	9, 29
7	25	F	560	Caesarean section^1^		Surgical NEC, PVL	No	16, 26
8	27	M	940	Caesarean section^2^		Medical NEC	No	16, 30
9	27	M	640	Caesarean section^3^		NEC totalis	Yes	16, 23, 35
10	26	M	880	Caesarean section^1^			No	13, 27
11	24	M	500	Caesarean section^2^	CONS		Yes	23

Indication for Caesarean section: 1-Breech presentation, 2-pregnancy induced hypertension, 3-fetal bradycardia.

CONS-coagulase negative staphylococcus, SVT-supraventricular tachycardia, PDA-patent ductus arteriosus, NEC-necrotizing enterocolitis, PVL-periventricular leukomalacia.

**Table 2:** Clinical features.

Medical Intervention	Days (Mean)
Duration* of antibiotics	15
Duration* of nystatin	21
Initiation of feeding**	9
Cumulative duration of feeding	23

*Duration reflects time prior to sample collection.

**Indicates day of life when started.

Among the cohort, five blood culture positive episodes of coagulase negative *Staphylococcus* (CONS) bacteremia were identified (Table 1). Three episodes of NEC occurred among enrolled infants with one episode, in infant 8, occurring during the sample collection period (Table 1; Information S2). There was one death during the study period due to a non-infectious medical complication.

### Eukaryotic microbial constituents of the ELBW enteric tract

**Figure 1:**
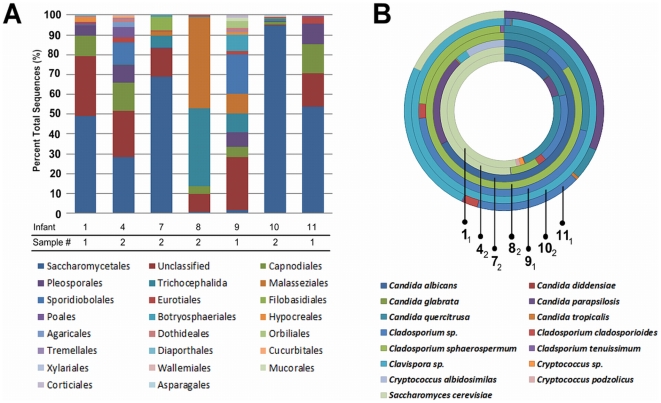
Lower eukaryotic sequence abundance. *Panel A:* Order level data per sample is shown as a percentage of the total sequences. *Panel B:* Predominant yeast genera and species. Best match genus-species estimates per infant's sample are shown. Where only a genus is indicated, species level data could not be confidently determined.

**Figure 2:**
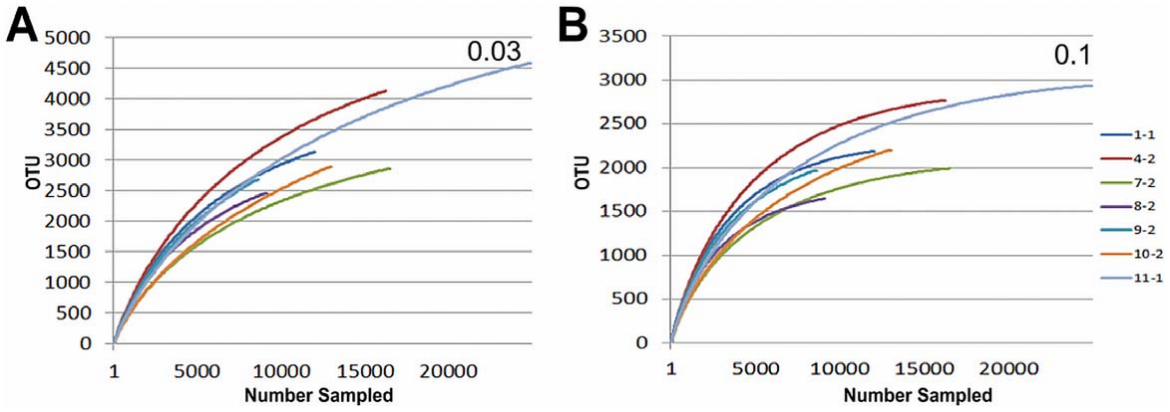
Rarefaction curves for lower eukaryotic sequences begin to plateau. Curves are identified by infant (first number) and sample number (second number). Phylogenetic cutoff distances for 0.03 (approximately species level) and 0.1 (approximately genus level) are shown in Panels A and B respectively.

Based on deep sequencing of eukaryote-directed ITS amplicons, sequences from fungal, metazoan and viridiplantae organisms were identified (Figure 1). Of the eleven infants, ITS amplicons were successfully amplified from seven infants. All experiments were run in parallel. Rarefaction curves of ITS amplicons begin to reach asymptotic levels, indicating sufficient depth of sequencing to account for most of the taxa amplified by ITS-targeted primers (Figure 2). *Saccharomycetales* was the most prevalent and abundant order, constituting 38.2% of amplicons in aggregate. Despite daily nystatin administration, a species-level examination of the yeasts present revealed sequences most similar to eight candidal species and three predicted cryptococcal species (Figure 1). Of the *Candida spp.* detected, six infants had fecal samples with abundant sequences corresponding to *C. quercitrusa* (Figure 1; Information S1). *C. quercitrusa* has been described in association with agriculturally-important fruit crops such as grapes and citrus but has never to our knowledge been identified in healthy humans or associated with infectious diseases [32], [33], [34].

Five infants had *Trichocephalida* sequences (E scores ranging from 0 to 1×10^−142^ and percent identity 90–98%) in the absence of clinical symptoms of infection (Figure 1). These 4,042 sequences were a best match at the genus level to *Trichinella*, a parasitic nematode (Information S1). Previously, ITS amplification has been shown to have excellent specificity for nematode identification and taxonomic classification [35]. Infant 8 had the greatest proportion of *Trichocephalida* sequences. On 16S rDNA analysis (described below), fecal genomic DNA from Infant 8 also had identifiable sequences corresponding to *Xenorhabdus*, a symbiont of some nematodes [36], providing further support for the presence of *Trichocephalida* in these infants.

### Bacterial constituents of the ELBW infant gut-associated microbiome

**Figure 3:**
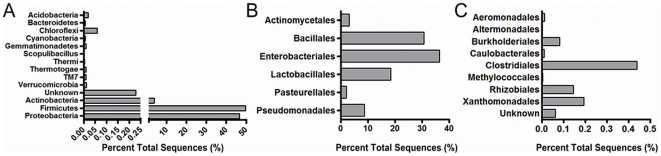
Bacterial phyla and orders in aggregate. Sequences from 16S rDNA were combined among all samples and analyzed in aggregate. Only taxa with greater than 50 sequences are shown. *Panel A:* Phyla-level sequence analysis. *Panel B*: Most-abundant orders (>0.5% of total sequences). *Panel C:* Low-abundance orders (<0.5% of total sequences).

In aggregate, over 95% of amplicons of the V6-8 region of the bacterial 16S rDNA coding sequences were classified at a phyla level as *Firmicutes* and *Proteobacteria*, with an additional eleven phyla being assigned at <1% abundance (Figure 3). Rare, low-abundance sequences were assigned to additional phyla not previously described in premature infants, including *TM7* and *Verrucomicrobia*. Even within the aggregated data, *Bacteroidetes* were extremely rare, suggesting that the gut of the ELBW infant does not support strictly anaerobic organisms.

**Figure 4:**
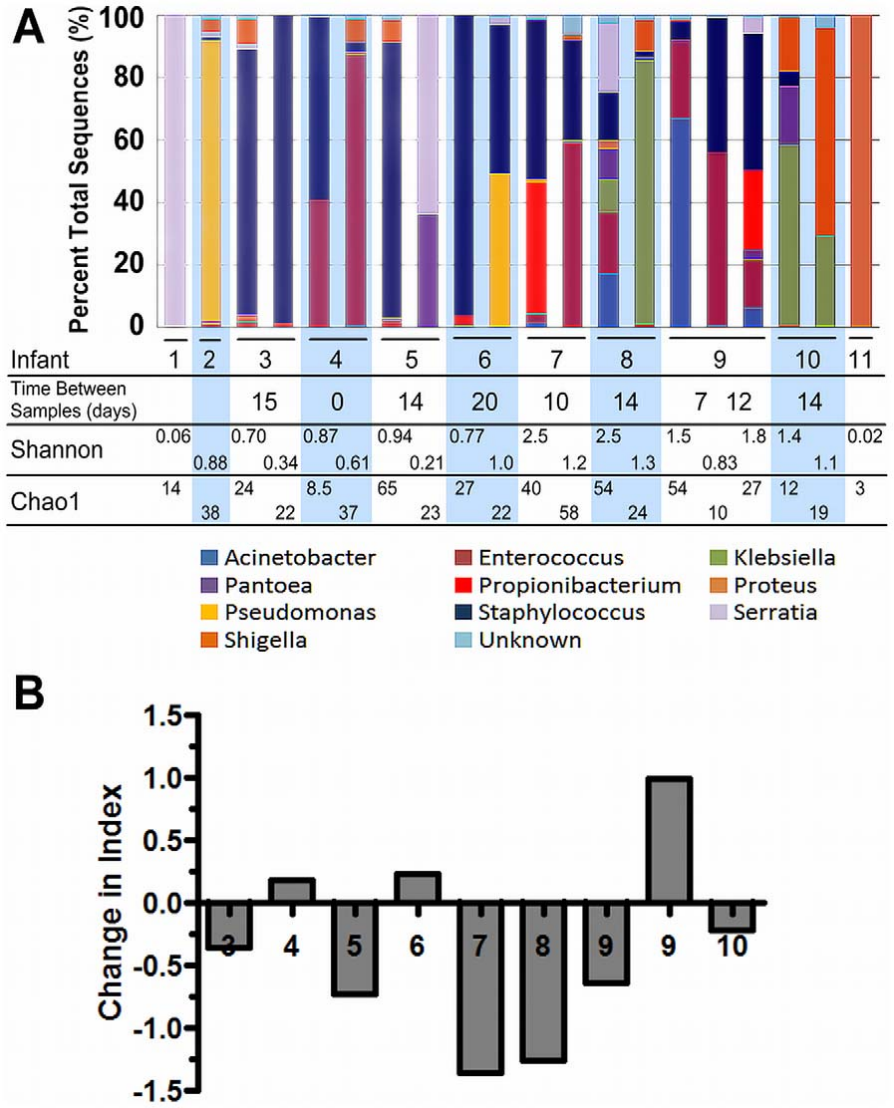
Bacterial genera by infant sample. *Panel A:* Percentages of the total sequences are shown for each sample. Shannon-Weaver Index (cutoff distance of 0.1) and Chao1 (cutoff distance of 0.1) are shown. *Panel B:* A graphical representation of the change in the Shannon-Weaver Index between samples per infant.

Among bacteria at the level of order, 99.7% of amplicons were identified in descending order of abundance as: *Enterobacteriales*, *Bacillales, Lactobacillales, Pseudomonadales, Actinomycetales*, and *Pasteurellales* (Figure 3). *Clostridiales* represented the most abundant order of known anaerobes but accounted for <0.5% of the total sequences (Figure 3). We identified a total of 61 genera, of which nine genera accounted for 94.7% of amplicons (Figure 4). Fifty-two genera represented less than 1% of all amplicons and the remainder were unclassified (Figure 4). We observed that 1–2 genera predominated in most of the fecal samples. Although shifts are observed between samples, the same genera often remain predominant even between samples collected several weeks apart and while off antibiotics (Information S2).

**Figure 5:**
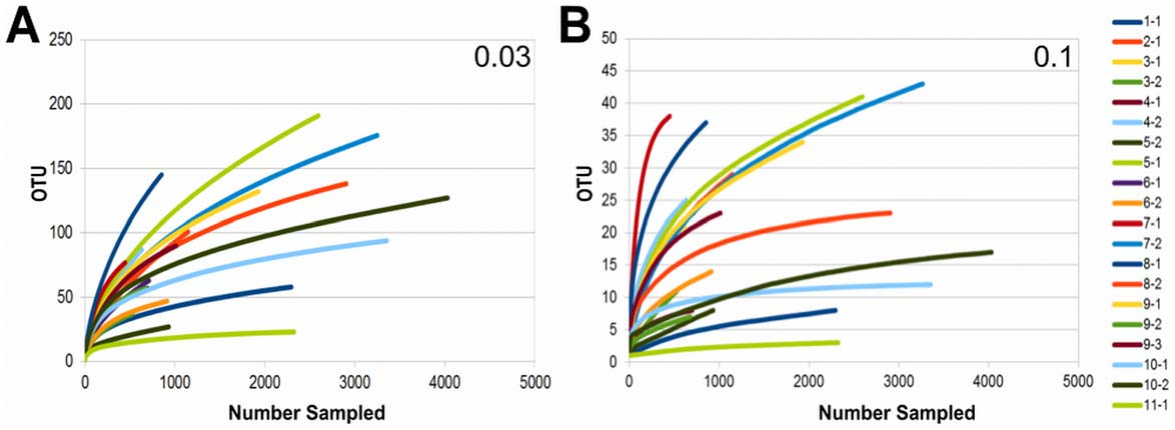
Rarefaction curves for 16S rDNA sequences. Curves are identified by infant (first number) and sample number (second number). Phylogenetic cutoff distances for 0.03 (approximately species level) and 0.1 (approximately genus level) are shown in Panels A and B, respectively.

To quantify community diversity, we calculated Shannon-Weaver indices [37] based on 16S rDNA sequences in each sample. Values ranged from 0.02 and 2.5 (distance cutoff = 0.1), with a mean of 1.02+0.69 (Figure 4), indicating a range of extremely low to moderate diversity. Among infants with multiple samples, the change in the Shannon-Weaver Indices between samples did not consistently increase (mean = 0.35+0.75), indicating that diversity was relatively static over time (Figure 4). Chao1 indices [38] ranged from 3 to 65, reflecting a range of taxonomic richness. In most individual samples, 1 to 2 genera comprised over 90% of the sequences as exemplified by Infant 6, Sample 1 (6-1). In this case, the Shannon-Weaver Index was lower than expected compared to the Chao1, reflecting the uneven bacterial population (Figure 4). Rarefaction curves of 16S rDNA sequences demonstrated adequate sampling in the majority of samples (Figure 5).

The composition of the microbiome is particularly notable in several cases of antibiotic administration and breast milk feedings. For instance, the fecal samples from Infant 1 had extremely low Shannon-Weaver and Chao1 indices 6 days after the administration of all antibiotics ended and after 11 days of breast milk feeding (Figure 4).

Notably, and in contrast to culture-based data in full-term infants, we found only 16 sequences among all of the samples corresponding to lactic acid fermenting bacteria despite exclusive breast milk feedings (*Lactobacillus sp.* or *Bifidobacterium spp.*) (Information S2). To confirm that the U968/L1401 primer set recognized these organisms, we obtained isolates of *Bifidobacteria sp.* and *Lactobacillus spp*. de-identified from the Duke Clinical Microbiology Laboratory. Genomic DNA was isolated using the same column genomic DNA preparation as was used for the fecal samples. Using the U968/L1401 primers in PCR amplification, amplicons of the expected product were produced, confirming that our amplification strategy would be expected to be sufficient to detect these genera were they present (data not shown).

### The metagenomes from 2 infant samples

To explore the viral and bacteriophage constituents of the microbiome, we used shotgun sequencing of MDA-derived total genomic DNA from 2 infants (samples 5-1 and 7-1). MDA was employed to overcome the low DNA content of the ELBW stool samples. However, this approach has known biases, including greater proficiency over low GC content, which is found in *Firmicutes*, the predominant taxa of both the samples analyzed. Also, in sample 5-1, 62% of sequences matched to a bacteriocin-like-peptide, likely a function of the random octamer primer. Due to these biases, the shotgun sequences may be best evaluated as qualitative and semi-quantitative data.

Approximately 71.5% of the shotgun-derived sequences were taxonomically assigned with E scores of <1e10^−5^ (“Classified”) (Table 3). The GC content in both samples was low, consistent with an abundance of gram-positive organisms, as revealed in directed 16S rDNA phylogenetic analysis (Figure 3). The average sequence length was >370 nucleotides (nt) from both samples, providing significant confidence in comparing the sequences to the SEED reference database [39]. As anticipated, sequences were primarily bacterial in origin. Among the bacterial sequences in each sample, *Firmicutes* predominated with rare *Bacteroidetes*. Sequences with a best match to Candidal species were identified. A small percentage of sequences originated from double-stranded and single-stranded DNA viruses. Single-stranded DNA virus sequences identified included bacteriophages S13, phiX174, and alpha 3. Double-stranded phage sequences included *Staphylococcus* Phage K, a *Caudovirales* species with a *Staphylococcus* host. Further analysis identified rare human adenovirus C sequences.

**Table 3:** Characteristics of sequences applied in metagenomics.

	5-1	7-2
**Classified**	70.32% (# 649,286)	73.08% (# 769,267)
**Unclassified**	29.69% (# 273,087)	26.95% (# 256,714)
**GC Content (Average; range)**	35% (5–70%)	25% (10–70%)
**Average sequence length**	370.4 nt	394.5 nt

Sequences for Infants 5 and 7 (samples 1 and 2 respectively) are shown. Classified sequences indicate the percentage and number with a phylogenetic assignment (based on the SEED database) at a score of<1e-5.

nt = nucleotides.

## Discussion

Overall, an initial analysis of organisms in the intestinal microbiome of ELBW infants revealed surprising fungal diversity, evidence of nematodes, and low to moderate bacterial diversity with nine genera accounting for the majority of sequences. Metagenomic analysis showed evidence of numerous bacteriophage as well as viral sequences. Examination of eukaryotic microbial sequences revealed environmental yeasts including *Candida albicans* despite universal prophylaxis with oral nystatin. Sequences closely corresponding to *Candida quercitrusa* were found, which previously has not been described in humans. In addition, sequences identified as *Trichinella,* a parasitic roundworm, were found in five infants.

Our analysis of the eukaryotic microbial constituents of the gut-associated microbiome in ELBW infants is the first of extensive depth and resolution. Due to varying abundance of fungi and parasites, ITS amplification was successful in only 7 of the 11 infants. Among the yeasts, we found DNA from pathogenic organisms typically sensitive to nystatin such as *C. albicans* as well as DNA sequences which correspond most closely to organisms with reduced susceptibility to nystatin such as *C. parapsilosis* and *C.tropicalis*. All of the infants received oral nystatin prophylaxis to decrease the risk of infection through the proposed mechanism of decontamination [40]. Although we cannot eliminate the possibility of nystatin resistant species, based on our results and culture-based data, oral nystatin may suppress *C. albicans* growth rather than eliminate the organism to prevent invasive disease [41], [42].

In addition to known pathogenic yeasts, the feces of six of the seven infants had sequences corresponding to *Candida quercitrusa*. This yeast has been described in fruit crops but does not have a known commensal or pathogenic relationship to humans, including neonates [33], [34]. *C. quercitrusa* has been used to control leaf blight through competitive exclusion, suggesting that the organism occupies microbial spaces at the expense of competing organisms [43]. We hypothesize that *C. quercitrusa* may be a benign yeast with unique tropism for the ELBW gut without virulence capacity, presenting an opportunity for its potential use in excluding other *Candida spp*. In order to understand the role of this organism in succession in the ELBW gut, future studies must explore *C.quercitrusa's* environmental sources, overall prevalence in the intensive care environment, and relationship with pathogenic yeasts in the gut of ELBW infants.

Surprisingly, we found that 5 of 7 infant samples contained sequences that matched within the genus *Trichinella* (e<−150), a parasitic roundworm, in the absence of clinical disease. Consistent with this unusual finding, sequences for the symbiont of nematodes, *Xenorhabdus spp.*, were identified in fecal genomic DNA from Infant 8 where the *Trichinella sp.* sequences were also most abundant[36]. PCR amplification would not be expected to differentiate DNA originating from *Trichinella* eggs or worms. We can only posit that the likely sources of acquisition for *Trichinella* would be parents, breast milk or hospital personnel. These unexpected results will need to be confirmed in larger cohorts of preterm infants, healthy term infants, and the intensive care setting.

Consistent with culture-based studies, we found limited bacterial diversity in the premature gut at the end of the first postnatal month. Nine genera accounted for∼95% of all the bacterial sequences. Because the predominant organisms we and others have identified also grow well in culture, prior studies using culture and lower-resolution molecular techniques provided a reasonable estimate of the major types of bacteria in the ELBW gut at single time points [15], [16], [17], [18]. However, true species richness and organisms at low abundance, which persist over time, can only be identified through deep sequencing. In order to understand the contribution of these less abundant bacteria in succession, infants must be sampled at defined intervals and analyzed with deep sequencing.

Using high-resolution techniques to examine the intestinal microbiome in preterm infants, limited data connects bacterial diversity with health outcomes. Wang, *et al* generated 16S rDNA clone libraries from infants with and without NEC and found lower diversity among the infants with NEC [21]. However, in our cohort, consistent with prior data from the same group, the one infant that developed medical NEC had a fecal sample collected 3 days prior, with a Shannon-Weaver Index of 2.51 above the average value of 1.02, suggesting that increased diversity, compared to peers, may not necessarily protect against NEC [20]. Our data also do not show a consistent increase in diversity over the first month of life.

When exploring the bacterial constituents using molecular techniques, previous studies found that *Proteobacteria* including *Enterobacteriacea* and *Pseudomonas* were most abundant in prior studies of the ELBW gut-associated microbiome [19], [20], [21]. In our cohort, *Staphylococcus spp*. and *Enterococcus spp.* were found in 75% of samples and were abundant in aggregate. In the case of *Staphylococcus spp.*, the genus was found in 95% of samples and accounted for >25% of the sequences among 10/20 samples (from 6 different infants). Furthermore, on the genera level, *Staphylococc*us maintained a significant proportion of the bacterial constituency over repeated sampling. In their cohorts, Wang, *et al.* and Millar, *et al.* also reported infants with abundant *Staphylococcus spp.* [14], [21]. However, in the Mshvildadze, *et al* study [20], the gut associated microbiome was dominated by *Streptococci sp,* an organism that we did not find among our infants. These significant differences may reflect environmental reservoirs in the respective intensive care units, differences in enteral nutrition, or varying antibiotic usage patterns.

While lactic acid fermenting bacteria have been found by culture, these organisms have not consistently been identified in the gut-associated microbiome of preterm infants using molecular techniques. These organisms, such as *Lactobacilli* and *Bifidobacteria*, modulate gut development and inflammation in cell culture and animal models [44], [45]. When used as a probiotic, they may also decrease the incidence of NEC and sepsis [46], [47], [48], [49]. Like data from Mshvildadze, *et al.* in preterm infants and Palmer, *et al.* in full-term infants [13], [20], our data failed to identify these organisms among infants, despite breast milk feedings. This is particularly notable for *Bifidobacteria* for which oligosaccharides in breast milk have been proposed to enhance their growth [50]. Thus, it is unlikely that lactic acid fermenting bacteria confer protection against invasive disease by competitive exclusion. If the goal of oligosaccharide supplementation of breast milk and formula is to increase colonization with lactic acid fermenting bacteria, this strategy may not be effective due to the relative absence of these organisms in primary succession of the preterm infant gut [51], [52].

While the gut is a known reservoir for invasive infection[9], not all infants who harbor potentially pathogenic organisms develop bacteremia. Other factors such as host immunocompetence, genetic susceptibility and severity of clinical course contribute to risk of invasive disease [53], [54]. And, though some researchers have hypothesized a microbial signature for NEC, no single organism or collection of organisms has consistently been linked to its development [55]. Consistent with our data, microbial diversity in the infant gut may not protect infants against development of invasive infection. Though diversity appears low at the genera level, subspecies differences among organisms may be important. We theorize that clones with enhanced pathogenicity may evade physiologic barriers and produce invasive disease. Within a microbial community, a clone without enhanced virulence may predominate and overwhelm other members. Or a pathogenic clone may be minor player in an unhealthy environment which does not restrain its growth and invasive capacity. Thus, future work will require subspecies-level analysis with temporal-spatial tracking of microbial constituents using in-depth environmental surveys to understand the sources of acquisition of specific clones that ultimately predominate in the microbiomes of individual infants and either cause or compete against infection.

As a first step in exploring the metagenome of the premature infant, we performed shotgun sequencing of genomic DNA amplified by MDA. Phylogenetic analysis of the genomic DNA confirmed the findings of 16S rDNA sequences, a predominance of *Firmicutes* and rare *Bacteroidetes*. The identification of Candidal species using MDA and ITS amplification corroborates its presence. In addition, the data suggest the presence of bacteriophage similar to previous research [22]. Furthermore, we have identified human adenovirus in the early ELBW enteric microbiome. The contributions of phage and viruses to the overall gut ecology will be a subject of future studies.

In conclusion, our goal was to broadly explore the range of microbes forming the ELBW gut microbiota during the critical first window of development. By the end of the first postnatal month, the ELBW microbiome is moderately diverse, but a small number of organisms are disproportionately over-represented, principally bacteria, among a complex milieu of yeasts and environmental molds. The first evidence of possible exposure to a parasitic organism, *Trichinella sp*., among ELBW infants in an intensive care unit in an industrialized nation, must be replicated in a separate cohort. Additionally, bacteriophage and viruses are present in the microbial communities that we observed. We hypothesize that these findings suggest that ELBW are subject to greater environmental exposure and acquisition of microbes than previously thought. Future studies must determine the sources of microbes that colonize the infants and elucidate how the widely variable microbiomes affect health outcomes in very premature infants, including invasive infections, necrotizing enterocolitis, growth, neurocognitive development, and immunity.

## Materials and Methods

### Clinical cohort

Eligible participants included ELBW infants (birthweight<1000 g) admitted to the Duke University Intensive Care Nursery. Each ELBW infant remained in a temperature and humidity controlled isolette during the study period. As part of standard clinical procedure, all health care providers wore gloves when handling ELBW infants for the first two postnatal weeks. Infants with congenital anomalies, predictable terminal conditions, or gastrointestinal tract anomalies were excluded from the study. After informed consent was obtained from parents, stool samples from the first postnatal month were transferred from diapers into sterile vials and stored at −80^°^C for processing.

### Ethics Statement

The Duke Institutional Review Board approved all study protocols (Pro 00000012). Samples and clinical information were obtained after informed, written consent by the study subjects*'* legal guardians.

### Stool genomic DNA extraction

Total stool genomic DNA was isolated using the Zymo Research Soil Microbe DNA Kit for all experiments except the metagenomic experiments that used Whatman FTA filter card technology [56]. Whatman FTA filter cards are designed for long term storage of nucleic acid at room temperature. For the Whatman FTA filter cards, stool was rolled onto one quadrant of an individual card with a sterile swab and allowed to dry overnight. Three to four 3.5-mm punches were made in the FTA cards using a hole punch pretreated with RNase Away followed by ethanol and flame sterilization of the punch device to ensure elimination of contaminating nucleic acids. The discs were soaked in 1% Triton X-100 in 1 X Tris-EDTA (TE; 10 mM Tris-HCl, 1 mM EDTA, pH 8) with 1% proteinase K for 15 minutes at 42°C. Next, the discs were placed onto a custom-designed vacuum filter unit washed three times with 1X TE made with diethylpyrocarbonate-treated water under suction. A final rinse using molecular biology-grade isopropanol was performed. The discs were dried at room temperature, and the genomic DNA was eluted from the discs into 1xTE by heating at 95°C for 5 minutes. Eluted samples were stored at −80°C until later use. Blank punches were performed in parallel for quality assurance that there was no contamination of the purification process.

### Amplification of bacterial rDNA and fungal ITS

Amplification of fungal and parasitic sequences was performed using ITS3 (GCATCGATGAAGAACGCAGCnbsp;) and ITS4 [TCCTCCGCTTATTGATATGC) [57], with the addition of 454 GS-FLX Titanium Primers A and B sequences from genomic DNA in 25 µl reactions performed in triplicate. Thermocycler conditions consisted of initial denaturation at 94°C x 5 minutes, amplification with 35 cycles of 94°C x 1 minute, 50°C x 1 minute, 72°C x 1 minute. PCR was completed with a 72°C cycle for 10 minutes followed by a 4°C dwell.

Amplification of 16S rDNA bacterial sequences from genomic DNA was performed using a U968 (AACGCGAAGAACCTTAC) and L1401 (CGGTGTGTACAAGACCC) primer set to amplify the V6-V8 region of 16S ribosomal DNA [58] Roche 454 GS FLX Titanium Primer A and B sequences and, where applicable, MID key sequences were added to the respective primers. Thermocycler conditions consisted of initial denaturation at 95°C for 3 minutes followed by 10 cycles of 95°C x 15 seconds, 62°C x 30 seconds decreasing temp 1°C every cycle, 72°C x 30 seconds followed by 25 cycles of 95°C x 10 seconds, 52°C x 30 seconds, 72°C x 30 seconds. Two independent amplification reactions were pooled for sequencing at the Duke IGSP Genome Sequencing & Analysis Facility using Roche 454 GS-FLX Titanium technology. Purified genomic DNA from *E.coli* strains UTI89 and MG1655 were used as positive controls [59], [60].

### Metagenomic shotgun sequencing

Using three 3.5 mm-diameter discs per sample, approximately 5–20 ng of DNA was then subjected to whole genome amplification as follows. Genomic DNA was denatured with 200 mM HCl followed by neutralization with 200 mM Tris-HCl, pH 7.5. Trehalose was added on ice. This mixture was combined with 10 µl of 10×phi29 buffer (Fermentas), 25 mM dNTP mixture, 500 µM random octamer primer [61] , Trehalose 1.5 M (final; and 1 µL of 100 x acetylated BSA (NEB). This mixture was incubated for 16 hours at 30°C and heated for 20 minutes at 70°C.

For all techniques described, negative controls included amplification reactions without template and eluate from empty genomic DNA isolation column preparations. Gel electrophoresis and ethidium bromide staining was used to verify the presence and absence of amplicons, plus their size and integrity. The amplicons from experimental samples were discarded if amplicons were visualized from negative control reactions.

### ITS amplicon analysis

Successful amplification using ITS3/ITS4 primers from genomic DNA was achieved in seven of the eleven infants. Experiments were run in parallel. There was no cross contamination or difference in lab protocol. Primers were removed *In Silico* from all 454 amplicons. We constructed a 672,000 sequence database of ITS region 1 and 2 from NCBI. A total of 87,560 454 ITS amplicons were then BLAST-searched against this ITS database to determine sequence identity. Per sample, the average number of sequences was 4919 [range 2157–11656] with an average read length of 280 nt [range 61–381].Only ITS sequence matches with greater than 75% identity were kept for further analysis. The highest e-value was kept for each sequence meeting the above criterion. In the event of a 454 sequence having multiple database sequence matches with the same e-value, the first match reported was used. Taxonomic assignments based on NCBI taxonomy were then compiled for graphical representation at the Phylum, Class, Order, Family, and Species level with the number occurrences tabulated. In addition, to analyze the quality of the matches , all sequences with their corresponding e-values and percent identity in the *Trichinella* genus were collected to construct frequency distribution graphs.

### 16S rDNA amplicon analysis

A total of 1.1×10^5^ sequences between 150–450 nt were obtained from the stool samples of the eleven infants. Sequences were processed and analyzed through a pipeline entailing pyrotagger [pyrotagger.jgi-psf/org/;] [62], Mothur tools [63], and RDP Classified [64]. Pyrotagger eliminates sequences with Phred values <27, performs dereplication, and clusters non-redundant sequences using a 97% sequence identity threshold, approximately species level distance [62]. Groups of sequences from independent samples were processed using subprograms within Mothur for confirmation of taxonomic assignments as follows [63]. Sequences were trimmed of primer and mid tags while retaining only sequences with a minimum size of 150 nt, q-average of 25, and maximum homopolymer tolerance of 10. Unique sequences were aligned against the greengenes reference alignment database (4,938 bacterial and archaeal sequences) followed by filtering to remove empty columns. Sequences were compared to the RDP database [64], verifying the relative proportions of sequences for the predominant organisms identified through Pyrotagger. Chimera identification and removal was performed using Uchime as implemented in Mothur. After these processing steps, the average number of sequences per sample was 1718 [range 450–4031]. Phylogenetic distances were determined using the functions dist.seqs and cluster functions in Mothur. Rarefaction curves, Shannon-Weaver Index, and Chao1 Index [38] were determined using the functions rarefaction.seqs and summary.single on the high quality sequence data pre-processed in Mothur. Cutoff distances of 0.1 and 0.03 (90% and 97%) were used as indicated for phylogenetic distances. At the genus level, E scores ranged from 1×10^−95^ to zero, providing confidence in the genera assignments.

### Metagenome analysis

For analysis of the metagenome, sheared MDA-derived DNA was subjected to 454 Titanium sequencing, yielding a total of 1,948,354 sequences. The sequences were analyzed using MG-RAST and the SEED database for phylogenetic assignment of the sequences [39].

## Supporting Information

Information S1 **E-score and percent identity for** ***Trichinella pseudospiralis and Candida quercitrusa*** **sequences**. ***Panels A, C:*** Histogram of E-score distribution of 454 Titanium sequences that matched *Trichinella pseudospiralis* and *Candida quercitrusa* ITS2 regions, respectively. ***Panels B, D:*** Percent identity frequency distribution of 454 Titanium sequences that matched *Trichinella pseudospiralis* and *Candida quercitrusa* ITS2 regions, respectively.
(DOC)

Information S2 **Diagram of feeding, antimicrobial administration, infection and sampling patterns.**
(DOC)
