# Supplementary material for: Beyond Bacteria: A Study of the Enteric Microbial Consortium in Extremely Low Birth Weight Infants
Source: PLoS One. 2011 Dec 8;6(12):e27858. doi: 10.1371/journal.pone.0027858 (PMC3234235; doi:10.1371/journal.pone.0027858)
Supplement: Information S1 — E-score and percent identity for Trichinella pseudospiralis and Candida quercitrusa sequences. Panels A, C: Histogram of E-score distribution of 454 Titanium sequences that matched Trichinella pseudospiralis and Candida quercitrusa ITS2 regions, respectively. Panels B, D: Percent identity frequency distribution of 454 Titanium sequences that matched Trichinella pseudospiralis and Candida quercitrusa ITS2 regions, respectively. (DOC) [file pone.0027858.s001.doc]

**Supporting information S1. E-score and percent identity for *Trichinella* *pseudospiralis and Candida quercitrusa* sequences**

**
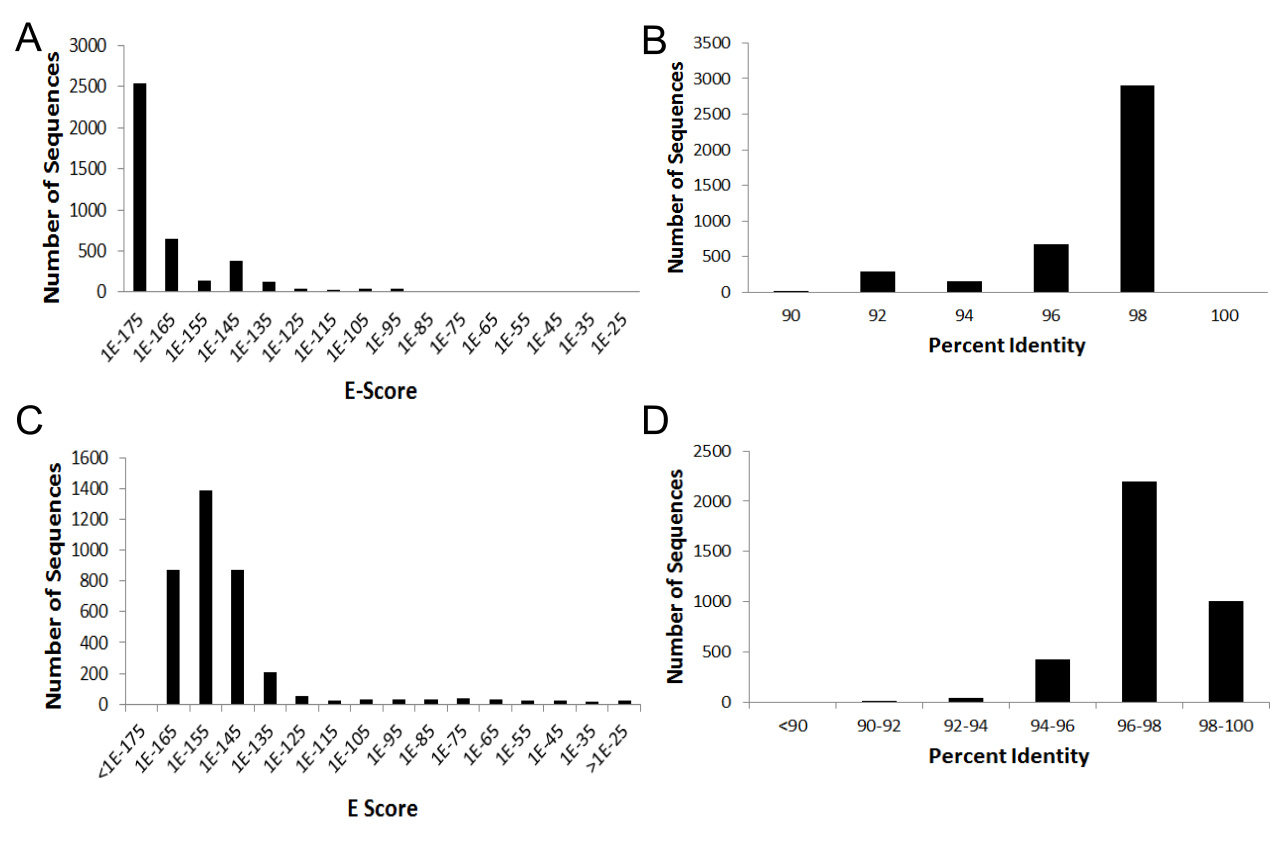
**
